# Association Between Vitamin D Deficiency, Malnutrition, and Systemic Inflammation in Advanced Colorectal Cancer: A Hospital-Based Cross-Sectional Study

**DOI:** 10.3390/nu18071059

**Published:** 2026-03-26

**Authors:** Daylia Thet, Chidchanok Rungruang, Nutthada Areepium, Nattaya Teeyapun, Tippawan Siritientong

**Affiliations:** 1 Department of Food and Pharmaceutical Chemistry, Faculty of Pharmaceutical Sciences, Chulalongkorn University, Bangkok 10330, Thailand; daliathet@gmail.com; 2 Department of Pharmacy Practice, Faculty of Pharmaceutical Sciences, Chulalongkorn University, Bangkok 10330, Thailand; chidchanok.r@pharm.chula.ac.th (C.R.); nutthada.a@pharm.chula.ac.th (N.A.); 3 Division of Medical Oncology, Department of Medicine, Faculty of Medicine, Chulalongkorn University, Bangkok 10330, Thailand; amprabbit@yahoo.co.th; 4 Center of Excellence in Metabolomics for Life Sciences, Chulalongkorn University, Bangkok 10330, Thailand

**Keywords:** 25-hydroxyvitamin D, albumin, colorectal cancer, malnutrition, vitamin D

## Abstract

**Background/Objectives**: Vitamin D deficiency and malnutrition may lead to poor outcomes in colorectal cancer (CRC) patients. This study aims to perform an integrative analysis of serum vitamin D, nutritional status, anthropometric parameters and biochemical profiles in advanced CRC patients. **Methods**: The study included 58 advanced CRC patients. Serum vitamin D levels were measured by a chemiluminescence immunoassay. Nutritional status was evaluated with the Mini Nutritional Assessment (MNA). Body composition profiles were assessed using a bioelectrical impedance analyzer, and handgrip strength was measured with a handgrip dynamometer. Biochemical and clinical parameters were retrieved from an electronic database. Correlation, regression and receiver operating characteristic (ROC) analyses were performed. **Results**: Abnormal nutritional status and vitamin D deficiency were diagnosed in 55.17% and 50.00% of patients, respectively. Sarcopenia was diagnosed in 29.31%. Serum vitamin D concentrations were negatively correlated with absolute neutrophil counts (ANC). MNA scores showed significant negative correlations with ANC, platelet count, alkaline phosphatase and carcinoembryonic antigen. In multivariable regression models, albumin remained statistically associated with both serum vitamin D levels (β 7.049; 95% CI: 1.686–12.413; *p* = 0.011) and MNA score (β 6.951; 95% CI: 4.623–9.278; *p* < 0.001). Furthermore, albumin showed exploratory performance in ROC analyses for malnutrition and vitamin D deficiency (AUC^ROC^ 0.814 and 0.725, respectively), which should be interpreted cautiously given potential overlap with MNA-defined nutritional status and the limited sample size. **Conclusions**: Vitamin D deficiency, malnutrition and systemic inflammation commonly co-occur and are closely interrelated in patients with advanced CRC. A comprehensive assessment of nutritional status in a CRC supportive care setting is recommended.

## 1. Introduction

Colorectal cancer (CRC) is one of the leading cancers with high incidence and mortality globally [1]. It has been estimated that CRC incidence will exceed 3 million new cases by 2050 [2]. According to the national cancer statistics reported in Thailand in 2020, CRC remains the third most common cancer and represents 11% of the total cancer burden [3]. In CRC patients, malnutrition is a common comorbidity that particularly leads to worse clinical outcomes, including longer hospital stays, increased adverse events, and higher mortality [4]. Malnutrition remains prevalent among individuals with advanced CRC due to tumor burden, systemic inflammation, treatment intolerance, and metabolic dysregulation. A recent meta-analysis reported that malnutrition affects about 47.80% of overall CRC patients globally, including 12.10% with severe malnutrition and 33.10% with moderate malnutrition [5]. Advanced stages of CRC are particularly associated with higher prevalence of malnutrition due to the physiological burden of the disease and side effects of treatments [6]. Poor nutritional status has been related to worse survival outcomes in CRC. A prospective study of metastatic CRC (mCRC) patients receiving first-line fluoropyrimidine in combination with monoclonal antibody-targeted therapies such as bevacizumab, cetuximab, or panitumumab showed that malnutrition was strongly associated with poorer survival outcomes [7]. Patients with malnutrition had significantly shorter progression-free and overall survival, underscoring the prognostic importance of nutritional status in advanced CRC. Studies have suggested that nutritional measures and inflammatory biomarkers may act as predictors of both short- and long-term clinical outcomes in patients with CRC. In particular, biomarkers such as albumin, which reflect both nutritional status and systemic inflammatory responses, have been reported to be associated with prognostic outcomes in CRC patients [7,8,9].

Vitamin D plays an important role in modulating tumorigenesis in several cancers, including CRC. Studies have shown better clinical outcomes among individuals with sufficient serum vitamin D levels [10,11]. Accumulating studies have reported that higher circulating vitamin D levels are associated with improved prognostic outcomes in advanced CRC patients undergoing chemotherapy [12]. In rectal cancer, circulating vitamin D levels have been associated with clinical outcomes, with patients having sufficient 25(OH)D (>50 nmol/L) showing a lower risk of metastasis and mortality [13]. This finding suggests a potential synergistic effect of vitamin D in combination with neoadjuvant cytotoxic therapy. Additionally, vitamin D exerts significant immunomodulatory functions. Previously, studies have indicated that vitamin D deficiency is associated with elevated inflammatory biochemical parameters such as white blood cells and neutrophils, potentially reflecting dysregulated immune function [14].

Given the association between malnutrition, systemic inflammation, and body composition changes in cancer patients, biochemical parameters such as serum albumin, inflammatory indices (e.g., neutrophil and lymphocyte counts), and tumor-related markers, including carcinoembryonic antigen (CEA), are crucial as they collectively influence disease outcomes. However, despite growing evidence, further studies are required to clarify the complex relationships between vitamin D status, inflammatory and nutritional biochemical parameters, and anthropometric profiles in CRC populations, particularly in Asian patients, where data remain sparse. Investigating these interactions will advance the understanding of vitamin D’s comprehensive role in CRC prognosis and may guide nutritional and therapeutic strategies.

Despite the growing evidence on the roles of vitamin D and nutritional status in CRC, the integrative evaluation of serum vitamin D, nutritional state, muscle mass, and biochemical markers in metastatic CRC is limited, especially in Thailand. Understanding the relationship between poor vitamin D status, malnutrition, systemic biomarkers, and tumor burden may offer clinically useful perspectives for CRC supportive care. Thus, this study aims to investigate the prevalence of vitamin D deficiency and malnutrition among patients with advanced CRC and to examine their associations with body composition, muscle strength and biochemical profiles. It was hypothesized that vitamin D deficiency would be associated with poorer nutritional status, unfavorable body composition, and biochemical profiles in patients with advanced CRC.

## 2. Materials and Methods

### 2.1. Study Design and Population

This cross-sectional study was conducted among advanced CRC patients enrolled between March 2025 and October 2025 at King Chulalongkorn Memorial Hospital, Bangkok, Thailand. Eligible patients were consecutively identified and approached at the oncology unit during routine clinical visits for chemotherapy. The study recruited patients with histologically and cytologically confirmed advanced CRC, defined as stage IV metastatic disease based on the tumor-node-metastasis (TNM) staging system, aged ≥18 years, who were scheduled to initiate the 1st cycle of chemotherapy at the hospital during the recruitment period. The study population included both newly diagnosed metastatic cases and patients with recurrent or progressive disease. Prior treatments before enrollment, including surgery, radiotherapy or systemic therapy, were permitted to reflect a real-world cohort of advanced CRC patients, rather than a strictly treatment-naïve first-line population. Patients with cognitive impairment or pregnant women were excluded. All eligible patients were informed about the study objective and invited to participate. Patients who agreed and provided written informed consent were enrolled in the study. Face-to-face interviews and anthropometric assessments were conducted by trained clinical pharmacists (D.T., C.R., and research team) following standardized procedures. The time taken for the data collection process of each patient, including the interview, anthropometric assessments, and blood sample collection, was about 60–120 min. The study followed the Strengthening the Reporting of Observational Studies in Epidemiology (STROBE) guidelines (Supplementary Table S1) [15]. The ethical approval was obtained from the Institutional Review Board (IRB) of the Faculty of Medicine, Chulalongkorn University (IRB No. 0662/67) in accordance with the Declaration of Helsinki.

**Figure 1:**
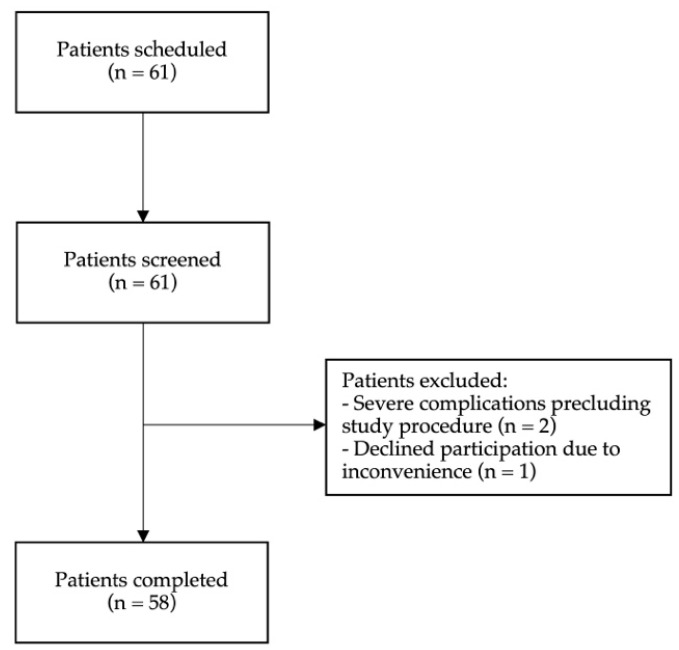
Flow diagram for participant recruitment.

The present study reports baseline cross-sectional data from an ongoing prospective cohort of patients with advanced CRC. Participants were recruited using a consecutive sampling approach during the predefined enrollment period. All eligible patients attending the oncology clinic between March 2025 and October 2025 who met the inclusion criteria were invited to participate. The final sample size (n = 58) reflected the number of patients recruited during this study period. A flow diagram summarizing the patient recruitment process, including exclusions and final inclusion, is presented in Figure 1.

### 2.2. Sample Collection and Measurement of 25(OH)D

Before chemotherapy was given to patients, blood samples were collected from each patient into serum clot activator tubes (Pro-coagulation tube, Xinle, Shijiazhuang, China) and allowed to clot before centrifugation at 3000 rpm for 10 min. The separated serum was aliquoted into microtubes and stored at −80 °C until analysis. Serum 25(OH)D levels were quantified via a chemiluminescence immunoassay (LIAISON^®^ 25 OH Vitamin D TOTAL Assay, DiaSorin, Saluggia, Italy), following the manufacturer’s instructions at King Chulalongkorn Memorial Hospital. Serum vitamin D status was classified as deficient (<20 ng/mL), insufficient (20–30 ng/mL), and sufficient (>30 ng/mL) [16].

### 2.3. Nutritional Status and Anthropometric Assessments

Nutritional status was assessed using the Mini Nutritional Assessment (MNA) prior to chemotherapy initiation. MNA evaluates dietary habits, anthropometric data, general information, and subjective assessments, including the patients’ self-perceived health status and self-perceived nutritional status [17]. Nutritional status was classified based on MNA scores as follows: normal nutritional status (24–30 points), at risk of malnutrition (17–23.5 points), and malnutrition (<17 points). MNA has been widely applied in oncology populations and has shown good sensitivity for identifying malnutrition in cancer patients, with evidence supporting its clinical relevance [18,19].

Pre-treatment body composition parameters, including body mass index (BMI), fat mass, fat% and muscle mass, were assessed using a bioelectrical impedance analyzer (BIA) (BC-418, Tanita, Tokyo, Japan). The appendicular skeletal muscle mass index (ASMI) value was calculated as the appendicular skeletal muscle mass of arms and legs (kg)/height (m^2^). According to the Asian Working Group for Sarcopenia (AWGS) 2025 Consensus Update, low appendicular skeletal muscle index (ASMI) was defined as <7.0 kg/m^2^ in males and <5.7 kg/m^2^ in females for individuals aged ≥65 years and <7.6 kg/m^2^ in males and <5.7 kg/m^2^ in females for those aged 50–64 years [20]. Handgrip strength (HGS) was evaluated using a handgrip dynamometer. Patients with sarcopenia were defined as having a low ASMI value together with low HGS of <28 kg in males and <18 kg in females (those aged ≥65 years) and <34 kg in males and <20 kg in females (those aged 50–64 years) [20]. Mid-arm circumference (MAC) and calf circumference (CC) were measured using a non-elastic tape.

### 2.4. Data Collection

Tumor size, treatment regimen types, histology, metastatic sites, comorbid conditions including hypertension, diabetes mellitus, and dyslipidemia, and other clinical factors were collected from electronic medical records. Patients received systemic chemotherapy in accordance with institutional treatment protocols, which were based on the National Comprehensive Cancer Network (NCCN) Clinical Practice Guidelines in Oncology for colon and rectal cancer. The prescribed first-line regimens were modified leucovorin + 5-fluorouracil (5-FU) + oxaliplatin (mFOLFOX6) and modified leucovorin + 5-FU + irinotecan (mFOLFIRI). Chemotherapy doses were calculated based on body surface area. In the mFOLFOX6 regimen, oxaliplatin (85 mg/m^2^) and leucovorin (200 mg/m^2^) were administered intravenously, followed by 5-FU given as a 400 mg/m^2^ intravenous bolus and 1200 mg/m^2^ continuous infusion. In the mFOLFIRI regimen, irinotecan (150–180 mg/m^2^) and leucovorin were administered, followed by 5-FU using a similar schedule. In some cases, targeted therapy such as bevacizumab was administered in combination with chemotherapy based on clinical indications. Laboratory biochemical parameters including albumin, hemoglobin, red blood cell count (RBC), white blood cell count (WBC), neutrophil percentage, lymphocyte percentage, absolute neutrophil count (ANC), platelet count, alkaline phosphatase (ALP), aspartate aminotransferase (AST), alanine aminotransferase (ALT), blood urea nitrogen (BUN), creatinine, estimated glomerular filtration rate (eGFR; calculated using the CKD-EPI equation), and CEA were retrieved from the hospital electronic medical records as part of the routine clinical laboratory testing. These laboratory parameters were obtained from tests performed within ±7 days of the nutritional assessment and blood sampling for serum 25(OH)D measurement prior to chemotherapy initiation.

### 2.5. Statistical Analysis

Normality test was performed. Descriptive data are presented as frequency with percentage and median with interquartile range (IQR). Mann–Whitney U test was used to assess the association of continuous variables across different groups. Chi-square or Fisher’s exact test was used to find the association between categorical variables. Spearman’s correlation was used to find the correlation between continuous variables. Receiver operating characteristic (ROC) curves and the corresponding areas under the curve (AUCs) were generated to evaluate the exploratory performance of nutritional and biochemical markers for identifying abnormal nutritional status (malnutrition + at risk of malnutrition) and vitamin D deficiency. The optimal cut-off values of test predictors were identified in compliance with the largest Youden’s index value. Univariate linear regression analysis was performed to find factors associated with serum vitamin D levels and nutritional status. Variables with *p*-value < 0.10 in univariate analysis were considered as candidates for multivariate models. Age and sex were included as potential confounding variables regardless of statistical significance due to their known influence on nutritional and metabolic status. For variables with missing values, analyses were performed using available-case (pairwise) analysis, in which all available observations were included for each specific analysis. Statistical analysis was performed using SPSS version 29.0. *p*-value of <0.05 was considered statistically significant.

## 3. Results

### 3.1. Participant Characteristics

A total of 58 advanced Thai CRC patients were included. The majority of participants had moderately differentiated histology. Thirty-two patients (55.17%) were female. The median age was 64.00 (56.00–70.00) years. Most patients had tumors primarily located in the colon (70.68%). The liver was the most common metastatic site (53.45%). The commonly prescribed chemotherapy regimens were mFOLFOX6 and mFOLFIRI. The median longest diameter of the tumor was 28.00 (11.50–75.00) mm. Some patients received targeted therapy such as bevacizumab. Common comorbidities were hypertension (41.37%), diabetes mellitus (18.96%) and dyslipidemia (37.93%). The characteristics of participants are presented in Table 1.

### 3.2. Prevalence of Malnutrition

The median MNA score was 22.75 (18.87–25.62), and 20 (34.48%) patients were at risk of malnutrition, while 12 (20.69%) were malnourished. Abnormal nutritional status (at risk of malnutrition + malnutrition) was observed in 55.17% of patients. The variables among patients with normal nutritional status (MNA ≥ 24) and those with abnormal nutritional status (MNA ≤ 23.5) were compared.

### 3.3. Anthropometric Characteristics

Overall, the median BMI, HGS, and ASMI values were 21.90 kg/m^2^, 19.05 kg, and 6.75 kg/m^2^, respectively. Low ASMI and low muscle strength were observed in 34.48% and 67.24%, respectively. Sarcopenia was found in 29.31% of our study. The anthropometric profiles between nutritional status groups are detailed in Table 2. BMI, MAC, and CC were significantly lower in patients with abnormal nutritional status (*p* < 0.001). Likewise, the abnormal nutritional status group had significantly lower profiles of ASMI (6.18 vs. 7.09 kg/m^2^, *p* = 0.006), fat mass (12.30 vs. 17.20 kg, *p* = 0.005), and HGS (16.65 vs. 22.70 kg, *p* = 0.014) compared to the normal nutrition group. In addition, malnourished patients were more likely to be sarcopenic than patients with normal nutritional status (*p* = 0.040).

### 3.4. Prevalence of Vitamin D Deficiency

The median serum 25(OH)D concentration was 20.30 (14.47–26.90) ng/mL, with 7 (12.07%) patients with sufficient, 22 (37.93%) with insufficient, and 29 (50.00%) with deficient serum vitamin D levels. Patients with abnormal nutritional status were more likely to have lower 25(OH)D levels than those with normal nutritional status, although statistical significance was not reached (*p* = 0.085) as presented in Table 3. Of note, in this study, we did not find a significant association between vitamin D status and sarcopenia or other anthropometric parameters, including HGS and body composition parameters.

Malnourished patients presented the lowest median vitamin D levels, while well-nourished patients had sufficient vitamin D levels (*p* = 0.018). Patients with malnutrition had significantly lower 25(OH)D levels than those with normal nutritional status (10.67 [7.37–24.45] vs. 21.25 [15.12–28.85] ng/mL).

### 3.5. Biochemical Profiles

As shown in Table 3, only well-nourished patients exhibited more favorable biochemical parameters, including platelet counts and alkaline phosphatase levels. Serum albumin levels were significantly lower in the malnourished group (*p* < 0.001). Similarly, patients with vitamin D deficiency had significantly lower levels of albumin (3.70 (3.10–4.00) g/dL vs. 4.20 (3.80–4.40) g/dL, *p* = 0.011). Regarding tumor markers, CEA levels were significantly higher in patients with abnormal nutritional status (*p* = 0.005). However, it should be noted that serum albumin may also be influenced by systemic inflammation, infection, or disease severity and therefore may not solely reflect nutritional status.

### 3.6. Dietary Information

Based on the dietary assessment of the MNA, 34.50% of patients reported a severe reduction in food intake, while 37.93% experienced unintentional weight loss of more than 3 kg during the previous 3 months. Regarding protein-rich food consumption, the majority of patients (86.21%) reported weekly intake of legumes or eggs, 67.24% consumed meat, fish, or poultry daily, and 58.62% reported the daily consumption of dairy products.

### 3.7. Correlation

Vitamin D levels showed positive correlations with MNA score (*p* = 0.013), serum albumin (*p* = 0.003) and lymphocyte% (*p* = 0.015), whereas negative correlations were observed with WBC (*p* = 0.036), neutrophil% (*p* = 0.008) and absolute neutrophil counts (ANC) (*p* = 0.027). No significant correlation was found between vitamin D and HGS or ASMI (*p* > 0.05). MNA scores were positively correlated with serum 25(OH)D, HGS (*p* < 0.001), ASMI (*p* = 0.001), albumin (*p* < 0.001), hemoglobin (*p* = 0.003) and lymphocyte% (*p* < 0.001), whereas negative correlations were observed with white blood cell (WBC) (*p* = 0.010), neutrophil% (*p* = 0.009), ANC (*p* = 0.015), platelet (*p* = 0.004), ALP (*p* = 0.011) and CEA (*p* = 0.011). The correlated factors of serum 25(OH)D and MNA scores are presented in Table 4.

### 3.8. Factors Associated with Serum Vitamin D and Nutritional Score

In univariate analysis, albumin and MNA score were found to have associations with serum 25(OH)D levels (Table 5). Regarding nutritional status, factors such as 25(OH)D, albumin, alkaline phosphatase, and tumor size were associated with MNA score. Variables showing potential associations in univariate analysis were entered into multivariable regression models. Due to the relatively small sample size, only a limited number of covariates were included in the multivariate models. In multivariate analysis, albumin remained statistically associated with both serum 25(OH)D (β 7.049; 95% CI 1.686–12.413; *p* = 0.011) and MNA score (β 6.951; 95% CI 4.623–9.278; *p* < 0.001) after controlling for age and sex.

### 3.9. ROC Analysis of Nutritional Markers

**Figure 2:**
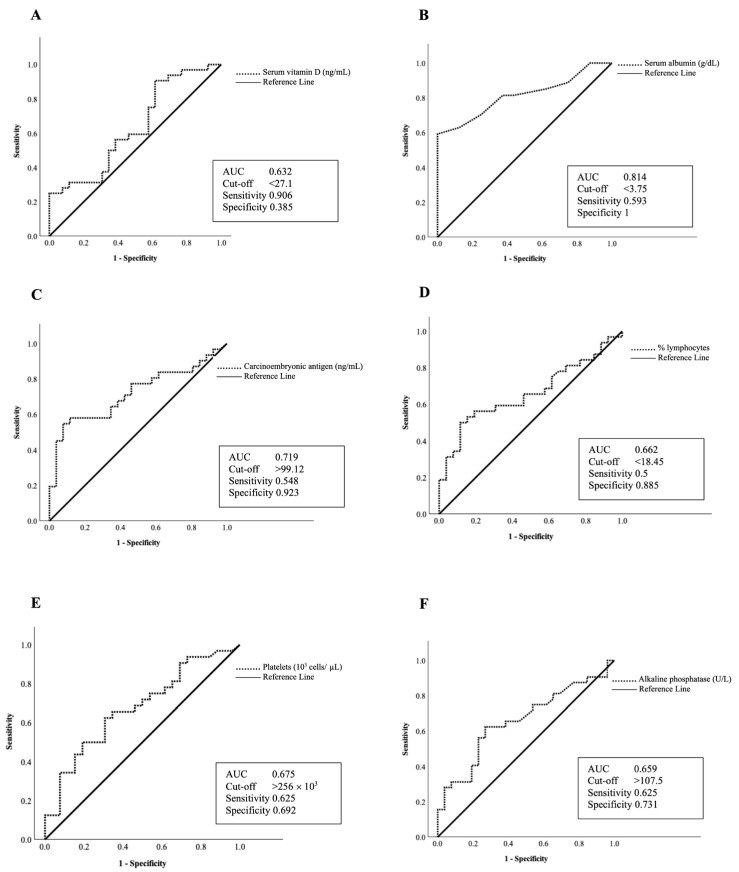
Receiver operating characteristic (ROC) curves of (**A**) serum vitamin D (*p* = 0.074), (**B**) albumin (*p* < 0.001), (**C**) carcinoembryonic antigen (*p* = 0.001), (**D**) lymphocyte% (*p* = 0.024), (**E**) platelets (*p* = 0.013), and (**F**) alkaline phosphatase (*p* = 0.027) for indicating risk of malnutrition in advanced CRC patients.

**Figure 3:**
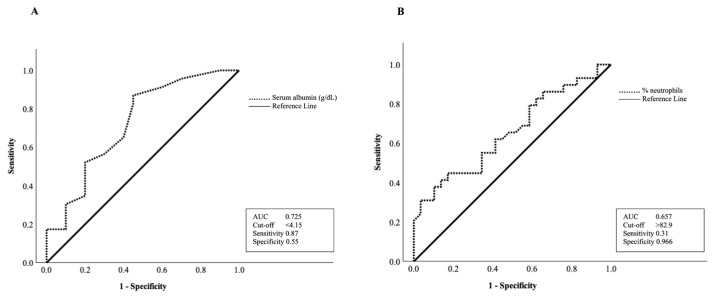
Receiver operating characteristic (ROC) curves of (**A**) serum albumin (*p* = 0.004) and (**B**) neutrophil% (*p* = 0.029) for indicating risk of vitamin D deficiency in advanced CRC patients.

The exploratory performance of biochemical parameters for identifying malnutrition and vitamin D deficiency is suggested according to the analysis based on the area under the ROC curve ≥ 0.5 as depicted in Figure 2 and Figure 3, respectively. In identifying malnutrition, several parameters demonstrated varying levels of performance. The AUC values were 0.814 for serum albumin (95% CI 0.688–0.939), 0.719 for CEA (95% CI 0.585–0.853), 0.662 for lymphocyte% (95% CI 0.521–0.802), 0.675 for platelets (95% CI 0.537–0.814) and 0.659 for alkaline phosphatase (95% CI 0.519–0.800). Although the statistical significance was not reached (*p* = 0.074), serum vitamin D also showed a borderline trend with an AUC of 0.632 (95% CI 0.487–0.777). Albumin demonstrated relatively higher AUC values within this cohort with a cut-off value of <3.75 g/dL (Figure 2). At this threshold, sensitivity was moderate (0.593) while specificity was high (1.000), indicating that a proportion of malnourished patients may not be identified when albumin is used alone. However, the observed specificity at this threshold should be interpreted cautiously, as it may reflect overfitting to the sample and may not be reproducible in other populations. For identifying vitamin D deficiency, only two parameters showed potential performance (Figure 3). Albumin (AUC 0.725; 95% CI 0.571–0.879) and neutrophil% (AUC 0.657; 95% CI 0.516–0.798) showed moderate to limited performance with cut-offs of albumin < 4.15 g/dL and neutrophil > 82.9%, respectively.

## 4. Discussion

### 4.1. Prevalence of Vitamin D Deficiency and Malnutrition in Advanced CRC

Our study showed serum vitamin D status in the advanced CRC population. Overall, 50% and 37.9% of patients had vitamin D deficiency or insufficiency, respectively. Similar patterns of low vitamin D status have been reported in the general Asian population. In a cohort of 1139 multi-ethnic Asian adults, 47.8% were vitamin D deficient and 28.4% were vitamin D insufficient [21]. Although that study was conducted in a non-cancer population, the high prevalence among the public highlights the underlying vulnerability to vitamin D insufficiency and deficiency across multi-ethnic Asians and provides important context for the high rates observed in our CRC cohort.

Malnutrition was also common in this population. Thirty-four percent were at risk of malnutrition, and 21% were malnourished. A similar high prevalence of malnutrition was reported in a hospital-based study in the Filipino CRC patients [22]. Based on the Subjective Global Assessment, 76.4% of patients in that study were moderately and severely malnourished, with more advanced disease stages markedly increasing the risk of malnutrition. Severe malnutrition was also associated with poor global health status and functional scores, underscoring its clinical relevance in the management of colorectal cancer.

### 4.2. Relationship Between Vitamin D Status, Nutritional Status, and Albumin

In our cohort, the burden of vitamin D deficiency was notably higher among patients with poor nutritional status, suggesting a potential association between micronutrient deficiency, systemic inflammation, and metabolic dysfunction. We observed that serum 25(OH)D levels were significantly correlated with nutritional and inflammatory parameters, supporting a potential role of vitamin D as an indicator of both nutritional and immunological status in patients with CRC. Similar observations have been reported in breast cancer populations, demonstrating the association of severe vitamin D deficiency with the development of cancer cachexia [23]. It highlighted the relationship between compromised vitamin D status and nutritional deterioration among cancer patients. In this context, the co-occurrence of vitamin D deficiency and poor nutritional status in the present study may reflect the underlying effects of cancer-associated cachexia and poor physiological reserve. Furthermore, the positive correlation between vitamin D levels and optimal nutritional status reinforces the link between malnutrition and vitamin D deficiency, particularly in older patients with advanced malignancies.

Importantly, one key finding was that serum albumin remained significantly associated with both serum 25(OH)D and nutritional scores. Albumin is a well-known prognostic marker reflecting nutritional status, systemic inflammation, and overall physiological deterioration in the cancer population [9]. However, albumin levels are susceptible to other factors, including liver function, fluid imbalance (e.g., edema) and overall disease burden. Therefore, albumin should be interpreted as a non-specific biomarker reflecting the combined effects of inflammation, nutritional status, and disease severity, rather than as a direct indicator of malnutrition or vitamin D status alone. In patients with advanced malignancies, decreased albumin levels may reflect not only nutritional impairment but also systemic inflammatory responses and metabolic alterations associated with cancer progression. The strong association between albumin and 25(OH)D in our study was consistent with a previous large cohort demonstrating that plasma 25(OH)D levels progressively decrease with decreasing albumin [24], which may partly reflect the association between inflammatory processes, nutritional status, and vitamin D metabolism.

Our ROC curve findings should be interpreted with caution. While previous evidence has reported albumin as a supportive parameter for nutritional assessment [25], its observed performance in this study likely reflects its sensitivity to systemic inflammation and disease burden rather than nutritional status alone. In our study, albumin showed a modest utility in identifying abnormal nutritional status; however, it should primarily be interpreted as a biomarker reflecting the combined influence of inflammatory and nutritional depletion. Nevertheless, the sensitivity observed at the identified cut-off was moderate, suggesting that albumin alone may underestimate the prevalence of malnutrition. Therefore, albumin should be interpreted as a supportive biochemical marker and used in conjunction with validated nutritional assessment tools, rather than as a standalone screening tool. Accordingly, the associations observed between albumin, vitamin D status, and nutritional scores should be interpreted as evidence reflecting a complex relationship between inflammation, nutritional status, and disease severity in advanced CRC. Although the specific cut-off values differed slightly, this variation is likely due to differences in population characteristics and clinical backgrounds. It should also be noted that albumin may be physiologically related to some components of nutritional assessment frameworks, such as MNA. Therefore, the observed association between albumin and MNA-defined nutritional status should be interpreted cautiously, as some conceptual and physiological overlap between these measures may exist.

### 4.3. Vitamin D, Nutritional Status and Biochemical Factors

Notably, WBCs and neutrophil percentages were higher in patients with vitamin D deficiency, supporting the immunomodulatory role of vitamin D, particularly in maintaining immune homeostasis. Previous studies have demonstrated inverse associations between vitamin D levels and inflammatory markers across diverse populations, including community-dwelling older adults and hospitalized patients [26,27]. In our cohort, vitamin D levels showed significant negative correlations with WBC and neutrophil%, reflecting the association between vitamin D deficiency and systemic inflammation.

Consistent with previous Asian literature, MNA scores showed positive correlations with serum albumin, HGS, and lymphocyte counts, supporting their role as a comprehensive indicator of nutritional and functional status [28,29]. More importantly, MNA scores were inversely correlated with WBC, neutrophil%, ANC, alkaline phosphatase, and CEA, suggesting that poor nutritional status may be associated with increased inflammatory burden and tumor activity.

### 4.4. Sarcopenia and Body Composition Findings

In this cohort, sarcopenia was found in 29.3% of participants, with malnourished patients more likely to be sarcopenic. This finding is consistent with a previous study in CRC populations, in which sarcopenia was observed in 15% of patients, and it has been associated with poorer nutritional status, lower BMI, reduced muscle quality, and lower serum albumin [30]. This finding highlights the close relationship between malnutrition and muscle wasting in CRC patients.

Nonetheless, no significant differences in body composition were observed across vitamin D categories in this population. Several assumptions may account for this finding. First, vitamin D deficiency may primarily affect muscle function rather than muscle mass. Second, body composition is influenced by multiple factors beyond micronutrient status, particularly in patients with advanced colorectal cancer. The lack of association between vitamin D levels and HGS or ASMI in our study reflects inconsistencies reported in previous Asian studies [31,32], suggesting that vitamin D may not be a key factor influencing muscle strength in older individuals with advanced-stage diseases. Third, the sample size may have been insufficient to detect small effect sizes.

### 4.5. Limitations and Strengths

It is important to note several limitations in this study. First, this was a single-center study with a relatively small sample size, which may limit the generalizability of the findings. Therefore, the results should be interpreted as exploratory rather than confirmatory. Due to the relatively small sample size, the multivariate models were adjusted only for age and sex to avoid model overfitting. Other potential clinical confounders, including disease stage, number and site of metastasis, performance status, and prior treatments, were not included in the models and should be considered in future studies with larger sample sizes. Although clinical variables related to tumor burden were collected, their potential influence on laboratory parameters and nutritional status was not evaluated in the current analysis. Tumor burden and disease severity may affect biochemical parameters such as albumin, inflammatory markers, and other laboratory indices independently of nutritional status. In addition, albumin is affected by multiple factors, including systemic inflammation, liver function, disease burden, and hydration status, limiting its specificity as a sole nutritional biomarker. The clinical utility of albumin as a standalone marker remains uncertain, and a causal relationship between albumin levels and clinical outcomes has not been clearly established [33]. The specificity value of albumin observed at the identified cut-off may reflect sample-specific characteristics and potential overfitting, precluding direct extrapolation to other populations. External validation in larger, independent cohorts is warranted to confirm its robustness. Moreover, the use of ROC analysis in this context should be interpreted with caution, as conceptual and physiological overlap with MNA-defined nutritional status may overestimate the apparent discriminatory performance of albumin. Therefore, these findings should be considered as hypothesis-generating regarding clinical implementation. Further studies should consider integrating these variables into multivariate analyses to better clarify their potential confounding effects. Moreover, we did not systematically collect information on the history of oral supplement use during patient recruitment, although none of the patients reported using specific vitamin D supplements. Standardizing vitamin D supplementation history may provide greater homogeneity among the participants in future research. Factors such as seasonal variation and sun exposure were also not assessed and may have influenced serum vitamin D levels. Serum 25(OH)D levels were measured only once prior to chemotherapy initiation. Follow-up measurements after chemotherapy treatments may provide a more comprehensive approach to evaluating its prognostic role. In addition, appendicular muscle mass was assessed using BIA, which may be less precise than dual-energy X-ray absorptiometry (DXA), the reference method for body composition measurement. Furthermore, BIA measurements may be influenced by hydration status, fluid imbalance, or edema, which may affect the accuracy of body composition estimation in patients with advanced diseases. However, BIA was used in this study because it is non-invasive and feasible in routine clinical practice. Nutritional status was assessed using MNA, which, although widely used and validated in oncology populations, is not a cancer-specific tool. Certain components of MNA, such as mobility and functional status, may partially reflect disease severity and general functional decline rather than nutritional status alone. Therefore, the interpretation of MNA-defined nutritional status should consider this potential overlap, particularly in patients with advanced CRC. Finally, as this was a cross-sectional analysis, causal relationships cannot be established. Despite these limitations, there are notable strengths. We performed a comprehensive nutritional assessment using a well-validated method alongside functional and biochemical parameters. The inclusion of both newly diagnosed metastatic and recurrent or progressive disease reflects a real-world advanced CRC population, enhancing the external validity of the findings. Crucially, by conducting all measurements prior to chemotherapy initiation, we minimized the confounding influence of acute treatment-related shifts in biochemical and nutritional status, ensuring a more accurate representation of the patients’ baseline inflammatory and metabolic profiles. Measurement of serum 25(OH)D using a standardized laboratory technique prior to chemotherapy initiation further reduces treatment-related confounding effects. The analysis of 25(OH)D, albumin, and other systemic inflammatory markers provides an integrative perspective on the association between the main parameters. Moreover, ROC analysis provides valuable exploratory insight into the comparative performance of nutritional markers.

### 4.6. Clinical Implications

Worse nutritional status and vitamin D deficiency are intercorrelated. As serum 25(OH)D may play a role in the treatment outcomes of advanced CRC patients, clinical monitoring and consideration of interventions would provide better management. Our study adds three clinically relevant contributions: (1) a high prevalence of vitamin D deficiency and malnutrition in a CRC cohort; (2) a correlation between vitamin D levels and nutritional indices and inflammatory markers; and (3) identification of serum albumin as a supportive parameter associated with both vitamin D and nutritional status. These findings support routine screenings for vitamin D deficiency and nutritional status and suggest the need for further studies on combined nutritional and vitamin D interventions in advanced CRC to make tailored nutritional counseling, supplementation, or referral to dietitians for better treatment tolerance and good quality of life in advanced CRC.

## 5. Conclusions

Low serum vitamin D levels are associated with malnutrition in patients with advanced CRC. Serum albumin was significantly associated with both vitamin D deficiency and malnutrition in this population; however, it should be interpreted as a non-specific biomarker influenced by multiple clinical factors rather than a direct indicator of nutritional status. A comprehensive evaluation of vitamin D and nutritional status may support personalized clinical assessment and further guide individualized nutritional care in various clinical settings.

## Figures and Tables

**Table 1:** Characteristics of participants (n = 58).

Characteristics	Total (n = 58)	Normal Nutritional Status (n = 26)	Abnormal Nutritional Status (n = 32)	*p*-Value
Age (years), median (IQR)	64.00 (56.00–70.00)	64.00 (48.50–69.00)	65.00 (56.25–71.00)	0.287
Female, n (%)	32 (55.17)	15 (57.69)	17 (53.12)	0.728
Family history of cancer, n (%)	26 (44.83)	9 (34.61)	17 (53.12)	0.191
Primary tumor site, n (%) - Colon - Rectum	41 (70.69) 17 (29.31)	19 (73.07) 7 (26.93)	22 (68.75) 10 (31.25)	0.778
Histology - Well differentiated - Moderately differentiated - Poorly differentiated - Other	17 (29.32) 30 (51.72) 5 (8.62) 6 (10.34)	4 (15.38) 18 (69.23) 1 (3.85) 3 (11.54)	13 (40.62) 12 (37.50) 4 (12.50) 3 (9.38)	0.065
Metastatic sites, n (%) - Liver - Lungs - Lymph nodes	31 (53.45) 28 (48.27) 14 (24.13)	14 (53.85) 13 (50.00) 4 (15.38)	17 (53.12) 15 (46.87) 10 (31.25)	0.956 0.813 0.221
Chemotherapy regimens, n (%) - mFOLFOX6 - mFOLFIRI - Others	21 (36.21) 34 (58.62) 3 (5.17)	6 (23.07) 18 (69.23) 2 (7.70)	15 (46.87) 16 (50.00) 1 (3.13)	0.170
Comorbidities, n (%) - Hypertension - Diabetes mellitus - Dyslipidemia - Chronic kidney disease	24 (41.37) 11 (18.96) 22 (37.93) 2 (3.45)	11 (42.31) 5 (19.23) 10 (38.46) 0 (0.00)	13 (40.62) 6 (18.75) 12 (37.50) 2 (6.25)	0.897 1.000 1.000 0.497

Mann–Whitney U test for continuous variables and Fisher’s exact test for categorical variables were used. Continuous data are presented as median (IQR). Categorical variables are presented as n (%).

**Table 2:** Anthropometric characteristics of participants.

Variables	Normal Nutritional Status (n = 26)	Abnormal Nutritional Status (n = 32)	*p*-Value
Nutritional score	26.00 (24.87–27.62)	20.00 (13.00–21.37)	<0.001
BMI (kg/m^2^)	23.85 (21.97–26.00)	20.40 (18.20–22.05)	<0.001
MAC (cm)	27.50 (25.00–28.00)	22.50 (21.00–25.00)	<0.001
CC (cm)	34.00 (32.00–35.25)	30.00 (29.00–33.00)	<0.001
Right leg muscle mass (kg)	6.75 (6.20–7.97)	6.40 (5.90–7.82)	0.340
Left leg muscle mass (kg)	7.05 (6.15–7.52)	6.40 (5.70–7.40)	0.118
Right arm muscle mass (kg)	2.05 (1.87–2.50)	1.75 (1.50–2.22)	0.013
Left arm muscle mass (kg)	2.05 (1.85–2.52)	1.75 (1.50–2.22)	0.013
ASM (kg)	17.95 (16.00–20.45)	16.50 (14.60–19.50)	0.099
ASMI (kg/m^2^)	7.09 (6.62–7.45)	6.18 (5.89–7.30)	0.006
Low ASMI, n (%)	6 (30.00)	14 (70.00)	0.095
Fat%	27.50 (23.27–37.77)	25.80 (19.20–29.80)	0.139
Fat mass (kg)	17.20 (14.45–22.10)	12.30 (10.50–18.15)	0.005
Fat-free mass (kg)	42.60 (39.62–48.87)	39.60 (35.37–44.85)	0.062
Basal metabolic rate (kcal)	1276.50 (1139.25–1361.50)	1091.50 (1003.75–1310.50)	0.005
Total body water (kg)	31.20 (28.97–35.80)	29.00 (25.87–32.87)	0.061
Visceral fat rating	10.00 (6.75–12.25)	8.00 (6.00–10.00)	0.139
Handgrip strength (kg)	22.70 (17.45–29.10)	16.65 (12.97–23.45)	0.014
Low handgrip strength, n (%)	14 (35.90)	25 (64.10)	0.090
Sarcopenia, n (%)	4 (23.53)	13 (76.47)	0.040

ASM, appendicular skeletal muscle; ASMI, appendicular skeletal muscle index; BMI, body mass index; CC, calf circumference; MAC, mid-arm circumference; Mann–Whitney U test for continuous variables and Fisher’s exact test for categorical variables were used. Continuous data are presented as median (IQR).

**Table 3:** Biochemical characteristics of participants by nutritional status.

Variables	Normal Nutritional Status (n = 26)	Abnormal Nutritional Status (n = 32)	*p*-Value
Serum 25(OH)D (ng/mL)	21.25 (15.12–28.85)	19.05 (10.06–26.42)	0.085
Albumin (g/dL)	4.20 (3.92–4.37)	3.60 (3.10–4.00)	<0.001
Hemoglobin (g/dL)	11.60 (10.52–12.25)	10.95 (10.02–11.85)	0.074
RBC (10^6^ cells/µL)	4.16 (3.69–4.74)	3.97 (3.58–4.26)	0.111
WBC (10^3^ cells/µL)	6.46 (4.99–8.81)	7.44 (5.58–10.58)	0.220
Neutrophil (%)	64.50 (55.75–71.12)	71.10 (55.85–83.50)	0.217
Lymphocyte (%)	26.15 (21.50–33.17)	18.60 (8.20–30.27)	0.035
ANC (10^3^ cells/µL)	4.24 (2.74–6.42)	5.16 (3.37–8.24)	0.302
Platelet (10^3^ cells/µL)	228.50 (164.00–288.00)	283.50 (209.75–360.00)	0.022
ALP (U/L)	92.50 (70.50–124.25)	134.50 (85.50–212.00)	0.038
AST (U/L)	31.00 (26.00–37.00)	38.50 (25.00–69.25)	0.090
ALT (U/L)	20.50 (16.50–28.25)	26.00 (17.00–36.75)	0.256
BUN (mg/dL)	14.00 (11.00–18.00)	12.50 (8.00–22.25)	0.797
Creatinine (mg/dL)	0.82 (0.61–1.06)	0.71 (0.53–1.08)	0.421
EGFR (CKD-EPI) (mL/min/1.73 m^2^)	88.34 (67.49–98.92)	90.96 (67.36–107.19)	0.601
CEA (ng/mL)	8.47 (3.49–31.54)	121.56 (11.29–1017.07)	0.005

ALP, alkaline phosphatase; ALT, alanine transaminase; ANC, absolute neutrophil count; AST, aspartate transaminase; BUN, blood urea nitrogen; CEA, carcinoembryonic antigen; EGFR, estimated glomerular filtration rate; RBC, red blood cell; WBC, white blood cell; Mann–Whitney U test was used. Data are presented as median (IQR). Sample sizes may vary slightly across variables due to missing data; pairwise analysis was applied. Reference ranges: serum 25(OH)D ≥ 30 ng/mL; albumin 3.5–5.0 g/dL; hemoglobin 12–16 g/dL (female), 13–17 g/dL (male); RBC 4.0–5.2 × 10^6^/µL (female), 4.5–6.0 × 10^6^/µL (male); WBC 4.0–10.0 × 10^3^/µL; neutrophils 40–75%; lymphocytes 20–40%; ANC 1.5–7.5 × 10^3^/µL; platelets 150–450 × 10^3^/µL; ALP 44–147 U/L; AST 10–40 U/L; ALT 0–40 U/L; BUN 7–20 mg/dL; creatinine 0.5–1.1 mg/dL (female), 0.7–1.3 mg/dL (male); eGFR ≥ 90 mL/min/1.73 m^2^; CEA 0–2.9 ng/mL. Reference ranges correspond to the institutional clinical laboratory standards.

**Table 4:** Spearman correlation of functional and biochemical factors with serum 25(OH)D and nutritional score.

Variables	Vitamin D	*p*-Value	MNA Score	*p*-Value
25(OH)D (ng/mL)	-	-	0.326	0.013
MNA score	0.326	0.013	-	-
Handgrip strength (kg)	0.117	0.382	0.439	<0.001
ASMI (kg/m^2^)	0.083	0.545	0.415	0.001
Fat%	−0.113	0.405	0.153	0.260
Fat mass (kg)	−0.080	0.557	0.351	0.008
Fat-free mass (kg)	0.202	0.136	0.342	0.010
BMR (kcal)	0.204	0.132	0.452	<0.001
Tumor size (mm)	−0.045	0.748	−0.203	0.145
Albumin (g/dL)	0.453	0.002	0.754	<0.001
Hemoglobin (g/dL)	0.099	0.460	0.387	0.003
RBC (cells/µL)	0.007	0.960	0.233	0.079
WBC (cells/µL)	−0.275	0.036	−0.334	0.010
Neutrophil (%)	−0.343	0.008	−0.340	0.009
Lymphocyte (%)	0.319	0.015	0.444	<0.001
ANC (10^3^ cells/µL)	−0.291	0.027	−0.319	0.015
Platelet (10^3^ cells/µL)	−0.193	0.147	−0.377	0.004
ALP (U/L)	−0.107	0.424	−0.333	0.011
AST (U/L)	0.069	0.610	−0.221	0.099
ALT (U/L)	0.009	0.947	−0.135	0.314
BUN (mg/dL)	0.215	0.138	0.109	0.457
Creatinine (mg/dL)	0.135	0.312	0.205	0.124
EGFR CKD-EPI (mL/min/1.73 m^2^)	−0.084	0.532	−0.180	0.181
CEA (ng/mL)	−0.119	0.380	−0.333	0.011

ALP, alkaline phosphatase; ALT, alanine transaminase; ANC, absolute neutrophil count; ASMI, appendicular skeletal muscle index; AST, aspartate transaminase; BMR, basal metabolic rate; BUN, blood urea nitrogen; CEA, carcinoembryonic antigen; EGFR, estimated glomerular filtration rate; MNA, Mini Nutritional Assessment; RBC, red blood cell; WBC, white blood cell. Spearman correlation coefficients were calculated using pairwise analysis. The number of observations may vary across variables due to missing data.

**Table 5:** Univariate and multivariate linear regression of associated factors of 25(OH)D levels and nutritional scores.

Variables	Univariate Analysis		Multivariate Analysis	
	Beta (95% CI)	*p*-Value	Beta (95% CI)	*p*-Value
Associated factors of 25(OH)D				
Age (years)	−0.003 (−0.254, 0.247)	0.978	0.009 (−0.279, 0.298)	0.949
Sex (Female vs. Male)	−2.131 (−7.447, 3.186)	0.425	−1.920 (−8.210, 4.369)	0.540
Albumin (g/dL)	7.314 (2.240, 12.388)	0.006	7.049 (1.686, 12.413)	0.011
CEA (ng/mL)	−0.001 (−0.003, 0.001)	0.363	-	-
Tumor size (mm)	−0.033 (−0.096, 0.029)	0.289	-	-
Nutritional score	0.712 (0.274, 1.150)	0.002	-	-
Associated factors of MNA scores				
Age (years)	−0.069 (−0.208, 0.070)	0.324	−0.048 (−0.155, 0.060)	0.375
Sex (Female vs. Male)	−0.624 (−3.615, 2.367)	0.678	−0.262 (−2.577, 2.054)	0.820
25(OH)D (ng/mL)	0.224 (0.086, 0.361)	0.002	0.044 (−0.079, 0.166)	0.472
Albumin (g/dL)	7.833 (5.922, 9.743)	<0.001	6.951 (4.623, 9.278)	<0.001
ALP (U/L)	−0.010 (−0.016, −0.004)	0.001	−0.003 (−0.008, 0.003)	0.292
Tumor size (mm)	−0.035 (−0.069, −0.001)	0.043	-	-

ALP, alkaline phosphatase; CEA, carcinoembryonic antigen; MNA, Mini Nutritional Assessment.

## Data Availability

The data that support the findings of this study are available from the corresponding author upon reasonable request. Due to privacy and ethical restrictions, the raw patient data cannot be shared publicly.

## Supplementary Materials

The following supporting information can be downloaded at: https://www.mdpi.com/article/10.3390/nu18071059/s1, Table S1: STROBE-nut: An extension of the STROBE statement for nutritional epidemiology.

## Abbreviations

ALP	Alkaline phosphatase
ALT	Alanine transaminase
ANC	Absolute neutrophil count
ASMI	Appendicular skeletal muscle mass index
AST	Aspartate transaminase
BMI	Body mass index
BMR	Basal metabolic rate
BUN	Blood urea nitrogen
AUC	Areas under the curve
CC	Calf circumference
CEA	Carcinoembryonic antigen
CRC	Colorectal cancer
EGFR	Estimate glomerular filtration rate
HGS	Handgrip strength
IQR	Interquartile range
MAC	Mid-arm circumference
mCRC	Metastatic colorectal cancer
MNA	Mini Nutritional Assessment
NCCN	National Comprehensive Cancer Network (NCCN)
RBC	Red blood cell
ROC	Receiver operating curve
STROBE	Strengthening the Reporting of Observational Studies in Epidemiology
WBC	White blood cell
